# Approximation of Composition and Temperature Dependent Heat Conductivity and Optimization of Thermoelectric Energy Conversion in Silicon–Germanium Alloys

**DOI:** 10.3390/e24101397

**Published:** 2022-10-01

**Authors:** Vito Antonio Cimmelli, Patrizia Rogolino

**Affiliations:** 1 Department of Mathematics, Computer Science and Economics, University of Basilicata, Viale dell’Ateneo Lucano, 10, 85100 Potenza, Italy; 2 Department of Mathematical and Computer Sciences, Physical Sciences and Earth Sciences, University of Messina, Viale F. Stagno d’Alcontres, 31, 98166 Messina, Italy

**Keywords:** composition graded materials, silicon–germanium alloys, composition-dependent heat conductivity, efficiency of thermoelectric systems, minimum of energy dissipated

## Abstract

We analyze the efficiency as thermoelectric energy converter of a silicon–germanium alloy with composition and temperature dependent heat conductivity. The dependency on composition is determined by a non-linear regression method (NLRM), while the dependency on temperature is approximated by a first-order expansion in the neighborhood of three reference temperatures. The differences with respect to the case of thermal conductivity depending on composition only are pointed out. The efficiency of the system is analyzed under the assumption that the optimal energy conversion corresponds to the minimum rate of energy dissipated. The values of composition and temperature which minimize such a rate are calculated as well.

## 1. Introduction

### 1.1. State of the Art

Silicon–germanium alloys of the type ${\mathrm{Si}}_{c}{\mathrm{Ge}}_{1-c}$, with composition c∈[0,1] changing inside the system, are extensively used in modern technology [1] as, for instance, in design of thermoelectric energy generators [2]. The thermoelectric efficiency of energy generators $\eta_{el}=\frac{P_{\mathrm{el}}}{{\dot{Q}}_{\mathrm{tot}}}$, with $P_{\mathrm{el}}$ the electric power output, and ${\dot{Q}}_{\mathrm{tot}}$ thermal power entering in the system, is an increasing function the so called figure-of-merit $Z=\frac{\epsilon^{2}\sigma_{e}}{\lambda }$, where ϵ is the Seebeck coefficient, $\sigma_{e}$ the electrical conductivity, and λ the thermal conductivity [3,4]. It is easily verified the the physical dimension of Z is the inverse of temperature, namely $K^{-1}$. Hence, since it is sometime convenient to work with non-dimensional quantities, some authors denote as figure-of-merit the quantity ZT, with T the temperature. Critical analysis of assigning the figure-of-merit is given in [5,6]. If one remains in the frame of linear thermodynamics, for a system whose two sides are constantly maintained at different temperatures $T_{h}$ (the hottest temperature) and $T_{c}$ (the coldest one), it can be proved that the maximum efficiency is [4]

(1) $$\eta_{max}=\eta_{C}\frac{1-1/\xi }{1+1/\xi }$$

wherein $\eta_{C}=1-T_{c}/T_{h}$ is the classical Carnot efficiency, and $\xi \equiv \sqrt{\mathrm{ZT}+1}$. Thus, the higher ξ, (and, hence, the smaller λ), the higher $\eta_{max}$. However, any strategy for the reduction in λ should take into account the results of phonon hydrodynamics, wherein it is assumed that the transport of heat is due to the phonons [7]. Phonons are quasiparticles, which in a solid crystal form a rarefied gas, whose kinetic equation can be obtained similarly to that of an ordinary gas. Phonons interact among themselves and with the crystal lattice through:

- Normal (N) processes, conserving the phonon momentum;
- Resistive (R) processes, in which the phonon momentum is not conserved.

The frequencies $\nu_{N}$ and $\nu_{R}$ of normal and resistive processes, respectively, determine the characteristic relaxation times $\tau_{N}=1/\nu_{N}$ and $\tau_{R}=1/\nu_{R}$. Purely diffusive heat transport takes over when there are many more R processes than N processes, i.e., when $\nu_{R}$ tends to infinity and $\tau_{R}=1/\nu_{R}$ tends to zero. If, instead, there are only few R processes and many more N processes, then $\tau_{N}=1/\nu_{N}$ tends to zero, and a wavelike energy transport (second sound propagation) may occur. The total relaxation time τ can be calculated by according to the Mathiessen rule $1/\tau =1/\tau_{R}+1/\tau_{N}$, while the thermal conductivity is given by $\lambda =\varrho c_{v}\tau {\bar{v}}^{2}/3$, where ϱ is the mass density, $c_{v}$ the specific heat per unit mass at constant volume, and $\bar{v}$ is the average of the phonons’ speed. Thus, a reduction in λ would produce a reduction of τ, i.e., an increment of the phonon scattering [7,8], with a consequent increment of dissipation. Thus, numerator and denominator in the expression of $\eta_{el}$ cannot be controlled independently, and one should look for reductions of λ which produce moderate increment of phonon scattering.

Materials with composition-dependent thermal conductivity are often used to enhance Z [2,9,10,11] since, by grading appropriately the stochiometry, λ can be reduced, achieving so a consequent increment of Z [4,7]. We also observe that a further improvement of Z could be obtained by increasing the product $\epsilon \sigma_{e}$ as function of *c*. At the moment we are not aware of the data concerning the dependency of ϵ and $\sigma_{e}$ on *c*, but such a task could be considered in future studies. Herein, we limit ourselves to take into account the dependency of ϵ and $\sigma_{e}$ on temperature reported in [12], and use three different values of those quantities for three different operational temperatures. Different physical quantities as, for instance, the electronic part of the thermal conductivity, could be considered as well. The role of such material parameter has been considered in [13,14]. Indeed, the problem under consideration depends on several parameters of different nature. Herein, in order to obtain applicable results, we limited ourselves to consider a simple but meaningful case.

The thermoelectric efficiency of graded systems has been investigated in Refs. [15,16,17,18,19], where the dependency of the performance of a thermoelectric energy generator as function of the two independent parameters *c* and *x*, with *c* as the composition of the system, and *x* as the square root of the mean value of the temperature gradient applied to its boundaries, has been investigated extensively. We used the square root of the mean value of the temperature gradient and not the temperature gradient itself, in order to obtain a more manageable expression for the rate of energy dissipated. The first independent parameter, i.e., the composition cannot be tuned externally, since *c* is fixed after manufacturing the system. The second parameter, instead related to the applied temperature gradient, can be tuned externally. We have pointed out that the analysis of those systems yields new information on how manufacturing homogeneous thermoelectric generators made by silicon–germanium alloys, by determining the composition and the temperature gradient which optimize their efficiency. It is worth observing that our aim in [15,16,17,18,19] was not to obtain explicit values of the efficiency for given independent parameters, because such values depend on several physical quantities which can be changed arbitrarily, such as, for instance, the geometry of the system and the external electric field. This fact motivated our choice of determining the values of the independent physical parameters which minimize the energy dissipated along the thermoelectric process, whatever is the value of such energy. One could wonder if the minimum of energy dissipated corresponds to the optimal efficiency of the thermoelectric process. Our answer is affirmative, and is based on the following considerations. In the following, we disregard all the losses introduced in the production of ${\dot{Q}}_{\mathrm{tot}}$ and in the management of the generated difference of electrical potential, and we focus only on the thermodynamic process inside a thermoelectric wire of length L. It consists in the generation of an electric potential after that an amount of heat per unit time ${\dot{Q}}_{\mathrm{tot}}$ entered the system. Such a heat produces dissipation by Joule effect which, in any point *z* of the system and at any time *t*, is represented by the rate of energy dissipated $E\left(c\left(z\right),\;x\left(z,t\right)\right)\equiv \bar{E}\left(z,t\right)$. Then, in a thermoelectric process of duration $t_{0}$, the total energy dissipated is given by

(2) $$E_{tot}=\int_{0}^{L}\int_{0}^{t_{0}}\bar{E}\left(z,t\right)dzdt$$

so that, being the integrand a positive quantity, the right-hand side of Equation (2) is minimum if, and only if, the integrand function is minimum in any point of the domain of integration [19].

We underline again that the previous analysis regards only the process of thermoelectric energy conversion inside the wire. A more complete analysis should include the dissipation due to the production of ${\dot{Q}}_{\mathrm{tot}}$, and that due to the transport and management of the obtained difference of electric potential, as well as the details of the processing parameters. However, such an analysis is outside the scopes of the present research, and is more pertinent to the field of engineering.

In order to make our investigation applicable to real cases, in [15,16,17,18,19] we used the experimental data on thermal conductivity of silicon–germanium alloys as function of the composition, at T=300K, T=400K, and T=500K [20,21,22]. From the disposition of the experimental points in the plane (c,λ(c)) it is evident that the thermal conductivity is very steep in the two regions close to c=0, and c=1. Indeed, around c=0, due to the small value of *c*, the alloy can be considered as doped Ge, while, close c=1, due to the high value of *c*, the alloy can be considered as doped Si. Thus, since doped crystalline semiconductors have reduced thermal conductivity with respect to the alloy, such a rapid decreasing is expected. Between those two steep parts of the curve, the experimental values of λ have small variation, so that λ presents a kind of wide minimum between the two dilute zones c∈[0,0.1] and c∈[0.9,1]. From the mathematical point of view, such a behavior is well represented by the sum of two exponential functions

(3) $$\lambda \left(c\right)=A^{\prime }e^{B^{\prime }c^{2}+D^{\prime }c}+E^{\prime }e^{F^{\prime }c^{2}+G^{\prime }c}$$

with $A^{\prime }$, $B^{\prime }$, $D^{\prime }$, $E^{\prime }$, $F^{\prime }$ and $G^{\prime }$ as unknown (non independent) parameters, to be determined by NLRM, under the constraints $\lambda \left(0\right)=\lambda_{\mathrm{Ge}}$ and $\lambda \left(1\right)=\lambda_{\mathrm{Si}}$. Thus, as first step, we have looked for a fitting curve represented by the Equation (3), and applied the following iterative procedure [23]:

- Starting from the disposition of the experimental data in the plane (c,λ(c)), we have assigned an initial estimated value of each parameter entering Equation (3);
- We have generated the curve defined by the initial values of the parameters;
- We have calculated the sum of the squares (i.e., the sum of the squares of the vertical distances of the experimental points from the curve);
- We have adapted the parameters in such a way that the curve was as close as possible to the experimental points;
- We have stopped the calculations when we have observed a negligible difference of results in successive iterations.

In this way, we have obtained the best-fit curve of the data as

(4) $$\lambda \left(c\right)=\varphi \left(M,N,P,Q\right)e^{Mc^{2}+Nc}+\gamma \left(M,N,P,Q\right)e^{Pc^{2}+Qc}$$

with *M*, *N*, *P*, *Q* four independent material parameters, and φ(M,N,P,Q) and γ(M,N,P,Q) two material functions of them. The constraints $\lambda \left(0\right)=\lambda_{\mathrm{Ge}}$ and $\lambda \left(1\right)=\lambda_{\mathrm{Si}}$ led to the following form of the functions φ(M,N,P,Q) and γ(M,N,P,Q)

(5) $$\begin{array}{c} \varphi \left(M,N,P,Q\right)=\frac{\lambda_{\mathrm{Si}}-\lambda_{\mathrm{Ge}}\;e^{P+Q}}{e^{M+N}-e^{P+Q}},\;\gamma \left(M,N,P,Q\right)=\frac{-\lambda_{\mathrm{Si}}+\lambda_{\mathrm{Ge}}\;e^{M+N}}{e^{M+N}-e^{P+Q}} \end{array}$$

Some more details of NLRM applied to obtain a fit of the experimental data are given in Ref. [16]. Therein, it is illustrated the method for minimizing the mean distance between the experimental points and the fitting ones. Of course, such a distance cannot be reduced to zero, and this error affects the coefficients *M*, *N*, *P*, *Q*, and, as a consequence, the function λ given in Equations (4) and (5). We expect that this error will influence also the optimal values of *c*, T, and λ calculated in Section 3.

In [15,16,17,18,19], we have studied the effects of the action of an electric field E on a graded ${\mathrm{Si}}_{c}{\mathrm{Ge}}_{1-c}$ wire of length L, crossed by an electric current i. The right-hand side (z=L), was supposed to be at the temperature $T_{h}$, while the left-hand side, (z=0), was supposed to be at the temperature $T_{c}$. We assumed that i was flowing uniformly from left to right, and that the heat rate ${\dot{Q}}_{\mathrm{tot}}$ was entering uniformly into the hot side of the system, giving rise to a heat flux q.

In our investigations, we applied the basic equations of thermoelectricity, whose physical meaning is discussed, for instance, in [4,24]. One of the key quantities in thermoelectricity is the form of the heat flux. Classically, the Fourier-like constitutive equation

(6) q=−λ∇T+Πi

where Π is the Peltier coefficient, and Πi is the additional heat flux due to the circulation of the electric current i [4,7,25,26] is postulated. However, heat transport theory is currently broadening its field of applicability since, owing to the miniaturization, new phenomenologies, beyond the classical Fourier theory of heat conduction, have been discovered [27,28]. Those new phenomena depend on the relationship between the mean free path of the heat carriers *ℓ*, and the characteristic dimension of the conductor L, expressed by the Knudsen number Kn=ℓ/L. Fourier’s law is valid when ℓ/L≪1, namely, for ℓ≪L. However, Kn can increase for a reduction in *L*, as in miniaturization technologies. Thus, when the mean free path of the heat carriers is comparable to the characteristic dimension of the conductor, i.e., Kn≃1, more complicated transport laws for the heat flux are necessary [19,27,28]. In the present investigation, the constitutive equation for q has been supposed to be

(7) $$q=-\nabla q\cdot l-\lambda \left(1-b\right)\nabla T+\Pi i$$

where l denotes a characteristic-length vector, and *b*, (<1), is a dimensionless physical parameter entering the effective thermal conductivity $\lambda_{eff}\equiv \lambda \left(1-b\right)$ [25,26]. In stationary situations, Equation (7) arises in thermomass (TM) theory [29,30] of heat transport. In a non-stationary case, the thermomass heat transport equation reads

(8) $$\tau_{\mathrm{tm}}\frac{\partial q}{\partial t}-\rho c_{v}\frac{\partial T}{\partial t}l+\nabla q\cdot l+\lambda \left(1-b\right)\nabla T+q=0$$

wherein $\tau_{\mathrm{tm}}$ is a relaxation time [25,26,29,30]. In thermomass description, the heat flux is generated by a gas of heat carriers, characterized by an effective mass density and flowing through the medium under the action of a thermomass–pressure gradient. This gas is made by massive quasi-particles of heat carriers, named thermons, which are nothing but the vibrations of the molecules generated by heating the conductor, with null rest-mass and dynamic mass which may be calculated from the Einstein’s mass–energy duality. In gases and liquids, the thermons are supposed to be attached to the molecules or atoms of the medium. In solids, the thermomass gas coincides with the phonon gas for crystals, attached on the electron gas for pure metals, or just both of them for systems in which the heat carriers are phonons and electrons. The physical parameters entering Equation (8) are [19]

$$\tau_{\mathrm{tm}}=\frac{\lambda }{2\gamma \rho c_{v}^{2}T}$$

with the dimensionless parameter γ being the Grüneisen constant,

$$b=\frac{q^{2}}{2\gamma \rho^{2}{\left(c_{v}T\right)}^{3}}$$

standing for a dimensionless number which is called thermal Mach number of the drift velocity relative to the thermal-wave speed in the heat-carrier collection, and

$$l=\frac{\lambda q}{2\gamma \rho c_{v}{\left(c_{v}T\right)}^{2}}$$

a characteristic-length vector. In fact, the physical dimensions of |l| are meters, as it can be directly inferred by the dimensional analysis of Equation (7). It characterizes the strength of the non-Fourier effects introduced by Equation (7) and, for conceivable values of q, attains values which are always much smaller than those of the mean-free path of the thermons [25,26].

Equation (7) is non-linear in the heat flux and also accounts for first-order non-local effects through the term ∇q·l. In [15,16,17,18,19], we applied Equation (7), since it contains the meaningful concept of effective thermal conductivity, accounting for the experimental evidence that the thermal conductivity is not independent of the heat flux. The constitutive equation for the current density is

(9) $$i=-\sigma_{e}\epsilon \nabla T+\sigma_{e}E$$

with E as the electric field, herein regarded as an external force applied to the system [4].

Finally, Equations (7) and (9) must be coupled with the energy-rate equation [4]

(10) $$\rho \frac{\partial u}{\partial t}=-\nabla \cdot q+E\cdot i$$

where *u* is the specific internal energy, and the quantity E·i is the rate of energy production due to the circulation of electric current. According to second law of thermodynamics, for such a system, the energy dissipated along the process, locally can be written as

(11) $$T\sigma_{s}=E\cdot i-\left(\frac{\nabla T}{T}\right)\cdot q\;\;\;$$

where $\sigma_{s}$ denotes the local entropy production.

Our analysis has been carried out under the hypotheses that $\lambda \left(c,T\right)≃\lambda \left(c,T_{h}\right)$, that both q and E depend only on the position on the longitudinal axis *z*, and that q and i are parallel. Then, by using Equation (11), together with the constitutive equations for the heat flux and for the electric current, after some manipulations the local rate of energy dissipated can be written as follows [16]:

(12) $$E\left(c,x\right)=\frac{i^{2}}{\sigma_{e}}+i\left[\epsilon \;-\left(\Pi -\bar{E}\;\;\bar{l}\right)/T_{h}\right]x^{2}+\left[\lambda \left(c\right)\left(1-b\right)/T_{h}\right]x^{4}$$

where $\bar{E}$ and $\bar{l}$ denote the mean values on the interval [0,L] of |E| and |l|, respectively, and $x\equiv \sqrt{\frac{T_{h}-T_{c}}{L}}$ is the square root of the mean value of the temperature gradient. By Equation (12), we infer that the effective thermal conductivity λ(c)(1−b) influences, in a meaningful way, the rate of energy dissipated. It is worth noticing that in deriving the expression (12) of the energy dissipated we did not use the second Kelvin relation Π=ϵT because when the heat flux is given by Equation (7), such a relation could no longer be valid, as proved in [31].

Then, under the assumption that the optimal thermoelectric energy conversion corresponds to the minimum of the function E(c,x), we have determined the couples $\left(c_{opt},\;x_{opt}\right)$ which minimize E(c,x) in different situations. Moreover, in correspondence of each minimum, we have obtained the value $\lambda_{opt}$ of the thermal conductivity. For the sake of illustration, in Table 1 are summarized the results obtained in Ref. [18].

To our best knowledge, there are not similar investigations in literature, so that we cannot compare the present results with pre-existing ones. Their experimental confirmation could follow by their possible application to design and manufacturing of thermoelectric energy converters.

### 1.2. The Present Research

Another parameter which can be externally controlled is the operational temperature, namely, the temperature of the conductor during the process. Thus, for the same system considered in Ref. [18], it seems important to obtain the couples $\left(c_{opt},\;T_{opt}\right)$ which minimize the energy dissipated, once *x* is fixed. To achieve that task, we need to recalculate the energy dissipated as function of *c* and T, at constant *x*. A direct inspection of Equation (12) suggests that this can be obtained if, and only if, we are able to express λ as function of *c* and T. On the other hand, since at different temperatures correspond a different phonon scattering, we expect a different behavior of λ with respect to the case of constant temperature. Such a hypothesis seems to be confirmed by Table 2, wherein it is shown the thermal conductivity of a pure Si and pure Ge wire of length L=3mm at the temperatures T=300K, T=400K, and T=500K [18].

We note a marked difference of the values of λ for the different temperatures. Thus, a dependency of λ on temperature must be taken into account. Since we have no experimental data of λ as function of temperature for Si−Ge wires of length L=3mm, as a first step we look for an approximation of it, in the neighborhood of the reference temperatures T=300K, T=400K, and T=500K. In more detail, we look for λ in the form

(13) $$\lambda \left(c,T\right)=\varphi \left(T\right)e^{m\left(T\right)c^{2}+n\left(T\right)c}+\gamma \left(T\right)e^{p\left(T\right)c^{2}+q\left(T\right)c}$$

where φ(T), γ(T), m(T), n(T), p(T), and q(T) are suitable temperature-dependent material functions. For m(T), n(T), p(T), and q(T), we use a series expansion up to the first order of the terms *M*, *N*, *P*, and *Q* in the exponents of Equation (2), with a suitable correction factor to be determined in order to keep the points of minimum of the temperature close to the reference temperature. For φ(T) and γ(T), instead, we use the zeroth order approximation given by Equation (4). We guess that, since the functions m(T), n(T), p(T), and q(T) enter the exponents appearing in the expression of λ(c,T), even a small variation of them will produce a sensible variation in λ, so that we expect a remarkable difference with respect to the isothermal case. This fact is in accordance with our hypothesis that variations in temperature influence remarkably the phonon scattering and, as a consequence, the thermal conductivity.

Thus, around T=300K, for instance, we write

(14) $$\begin{array}{ccc} & & m\left(T\right)=h_{1}m_{0}+h_{2}m_{1}\left(T-300\right) \end{array}$$

(15) $$\begin{array}{ccc} & & n\left(T\right)=h_{3}n_{0}+h_{4}n_{1}\left(T-300\right) \end{array}$$

(16) $$\begin{array}{ccc} & & p\left(T\right)=h_{5}p_{0}+h_{6}p_{1}\left(T-300\right) \end{array}$$

(17) $$\begin{array}{ccc} & & q\left(T\right)=h_{7}q_{0}+h_{8}q_{1}\left(T-300\right) \end{array}$$

where $h_{i},\;i=1,\ldots 8,$ are the correction factors we are looking for, and $m_{i}$, $n_{i}$, $p_{i}$, and $q_{i}$, i=0,1, are the coefficients of the expansion. The quantities $h_{i},\;i=1,\ldots 8,$ do not derive by a theoretical calculation, but are chosen empirically in order to obtain values of λ which remain limited for T ranging in the neighborhood of the reference temperatures. In future studies, we plan to investigate the possible existence of optimal values of them. Notice that we do not impose the conditions $h_{1}m_{0}=M$, $h_{3}n_{0}=N$, $h_{5}p_{0}=P$, and $h_{7}q_{0}=Q$, which would guarantee that for T=300K the fit in Equation (2) is recovered. We are aware that such a strategy could appear unusual at a first look. Our choice is motivated by the experimental evidence that, in materials in which λ depends on composition and temperature, there is a different rate of phonon scattering with respect to materials in which λ is insensitive to the temperature variation, so that we foresee different values of λ for the same composition. Our hypothesis is confirmed by numerical investigations, because the conditions $h_{1}m_{0}=M$, $h_{3}n_{0}=N$, $h_{5}p_{0}=P$, and $h_{7}q_{0}=Q$ do not produce either an acceptable fit or a minimum value of the heat conductivity. Anyway, we underline again that here we do not obtain any fit of λ, because we do not have data on its dependency on T for Si−Ge wires of length L=3mm. Herein, we obtain only a first-order approximation of λ as function of T, and investigate how such temperature dependency influences the thermoelectric efficiency. Such a situation will be analyzed in more detail in Section 3. Then, we do not impose any limitation to Equations (14)–(17), and look for correction factors which yield a physically acceptable approximation of λ. In this way, we obtain a dependency of λ on *c* which is qualitatively similar to that obtained in [18], but having different values. In future studies, we aim at exploring how much the numerical error influences such differences, and if we can reduce them by improving our approximation of λ.

The procedure illustrated above is also applied for the other two temperatures under consideration, by using different correction factors. In this way, we obtain an approximation of the function λ(c,T) which allows to express the rate of energy dissipated E as function of *c* and T. Then, we determine the conditions ensuring the optimal efficiency of the thermoelectric energy conversion by calculating the points of minimum of E(c,T). The plots of λ(c,T) for different temperatures are obtained as well.

The paper runs as follows.

In Section 2, we apply the approximation procedure described above to obtain the thermal conductivity of a wire of length L=3mm as function of *c* and T.

In Section 3, we study the first and second derivatives of λ(c,T) to calculate its minima around the temperatures under consideration. We prove that for each temperature there exists one, and only one, couple $\left(c_{opt},\;T_{opt}\right)$ which minimizes E(c,T). Moreover, we discuss the results in view of our approximation of λ and explain how they can be used in manufacturing thermoelectric energy converters.

## 2. Approximation of Thermal Conductivity

Following the way paved in Ref. [18], herein we study the effects of the action of an electric field E on a graded ${\mathrm{Si}}_{c}{\mathrm{Ge}}_{1-c}$ wire of length L, crossed by an electric current i. The right-hand side (z=L), is supposed to be at the temperature $T_{h}$, while the left-hand side, (z=0), is supposed to be at the temperature $T_{c}$. Moreover, i is flowing uniformly from left to right, and an amount ${\dot{Q}}_{\mathrm{tot}}$ of heat per unit time is entering uniformly into the hot side of the element, giving rise to a heat flux q.

In order to obtain an approximation of the heat conductivity, we use the values of the material constants *M*, *N*, *P*, and *Q* determined in [18]. Such values are quoted in Table 3.

Then, up to the second-order approximation of M(T), N(T), P(T), and Q(T), we can write

(18) $$\begin{array}{ccc} & & M\left(T\right)=m_{0}+m_{1}\left(T-300\right)+\frac{1}{2}m_{2}{\left(T-300\right)}^{2} \end{array}$$

(19) $$\begin{array}{ccc} & & N\left(T\right)=n_{0}+n_{1}\left(T-300\right)+\frac{1}{2}n_{2}{\left(T-300\right)}^{2} \end{array}$$

(20) $$\begin{array}{ccc} & & P\left(T\right)=p_{0}+p_{1}\left(T-300\right)+\frac{1}{2}p_{2}{\left(T-300\right)}^{2} \end{array}$$

(21) $$\begin{array}{ccc} & & Q\left(T\right)=q_{0}+q_{1}\left(T-300\right)+\frac{1}{2}q_{2}{\left(T-300\right)}^{2} \end{array}$$

and, consequently,

(22) $$\begin{array}{ccc} & & M\left(300\right)=m_{0}=M=4.8706 \end{array}$$

(23) $$\begin{array}{ccc} & & M\left(400\right)=m_{0}+100m_{1}+\frac{1}{2}m_{2}100^{2}=91.804 \end{array}$$

(24) $$\begin{array}{ccc} & & M\left(500\right)=m_{0}+200m_{1}+\frac{1}{2}m_{2}200^{2}=80.4998 \end{array}$$

By the relations above we obtain

(25) $$m_{0}=4.8706,\;m_{1}=1.36052,\;m_{2}=-0.00982376$$

Analogously, for B(T), D(T), and E(T) we obtain

(26) $$\begin{array}{ccc} & & N\left(300\right)=n_{0}=N=-3.76 \end{array}$$

(27) $$\begin{array}{ccc} & & N\left(400\right)=n_{0}+100n_{1}+\frac{1}{2}n_{2}100^{2}=-91.351 \end{array}$$

(28) $$\begin{array}{ccc} & & N\left(500\right)=n_{0}+200n_{1}+\frac{1}{2}n_{2}200^{2}=-80.0781 \end{array}$$

(29) $$n_{0}=-3.76,\;n_{1}=-1.37023,\;n_{2}=0.00988639$$

(30) $$\begin{array}{ccc} & & P\left(300\right)=p_{0}=P=109.452 \end{array}$$

(31) $$\begin{array}{ccc} & & P\left(400\right)=p_{0}+100p_{1}+\frac{1}{2}p_{2}100^{2}=4.416 \end{array}$$

(32) $$\begin{array}{ccc} & & P\left(500\right)=p_{0}+200p_{1}+\frac{1}{2}p_{2}200^{2}=4.0667 \end{array}$$

(33) $$p_{0}=109.452,\;p_{1}=-1.57379,\;p_{2}=0.0104687$$

(34) $$\begin{array}{ccc} & & Q\left(300\right)=q_{0}=Q=-108.953 \end{array}$$

(35) $$\begin{array}{ccc} & & Q\left(400\right)=q_{0}+100q_{1}+\frac{1}{2}q_{2}100^{2}=-3.3127 \end{array}$$

(36) $$\begin{array}{ccc} & & Q\left(500\right)=q_{0}+200q_{1}+\frac{1}{2}q_{2}200^{2}=-2.971 \end{array}$$

(37) $$q_{0}=-108.953,\;q_{1}=1.5829,\;q_{2}=-0.0105299$$

Although the procedure applied above allows to expand the functions M(T), N(T), P(T), and Q(T) up to the second order, as a first step of the application of the procedure, here we limit ourselves to insert their first order expansion into the constitutive equation of λ. Then, in view of Equations (18)–(21), we write

(38) $$\begin{array}{ccc} & & M(T)=4.8706+1.360522(T-300) \end{array}$$

(39) $$\begin{array}{ccc} & & N(T)=-3.76-1.370229(T-300) \end{array}$$

(40) $$\begin{array}{ccc} & & P(T)=109.452-1.57379(T-300) \end{array}$$

(41) $$\begin{array}{ccc} & & Q(T)=-108.953+1.582896(T-300) \end{array}$$

To proceed further, we use the values of pure Si and pure Ge [18], which are quoted in Table 2.

Hence, Equation (4) and Table 2 yield

(42) $$f\left(M\left(300\right),N\left(300\right),P\left(300\right),Q\left(300\right)\right)=\frac{\lambda_{\mathrm{Si}}\left(300\right)-\lambda_{\mathrm{Ge}}\left(300\right)\;e^{P(300)+Q(300)}}{e^{M(300)+N(300)}-e^{P(300)+Q(300)}}=15.5212$$

(43) $$g\left(A\left(300\right),B\left(300\right),D\left(300\right),E\left(300\right)\right)=\frac{-\lambda_{\mathrm{Si}}\left(300\right)+\lambda_{\mathrm{Ge}}\left(300\right)\;e^{M(300)+N(300)}}{e^{M(300)+N(300)}-e^{P(300)+Q(300)}}=62.4288$$

Finally, we obtain the following expression for λ in the neighborhood of T=300K

(44) $$\lambda \left(c,T\right)=15.5212e^{\left(1/10\right)c\left(-3.76-1.37023\left(-300+T\right)\right)+\left(1/10\right)c^{2}\left(4.8706+1.36052\left(-300+T\right)\right)}$$

$$+62.4288e^{\left(1/100\right)c\left(-108.953+1.5829\left(-300+T\right)\right)+\left(1/100\right)c^{2}\left(109.452-1.57379\left(-300+T\right)\right)}$$

wherein we have taken $h_{1}=h_{2}=h_{3}=h_{4}=1/10$ and $h_{5}=h_{6}=h_{7}=h_{8}=1/100$.

By the same procedure we obtain the following expressions of λ(c,T), around T=400K and T=500K, respectively.

(45) $$\lambda \left(c,T\right)=45.4922e^{\left(1/10\right)c\left(-91.351-0.38155\left(-400+T\right)\right)+\left(1/10\right)c^{2}\left(91.804+0.378097\left(-400+T\right)\right)}$$

$$+13.9278e^{\left(1/100\right)c\left(-3.3127+0.529907\left(-400+T\right)\right)+\left(1/1000\right)c^{2}\left(4.416-0.526927\left(-400+T\right)\right)}$$

(46) $$\lambda \left(c;T\right)=12.8393e^{\left(1/10\right)c\left(-2.971-0.523076\left(-500+T\right)\right)+\left(1/10\right)c^{2}\left(4.066+0.51993\left(-500+T\right)\right)}$$

$$+35.2434e^{\left(1/10\right)c\left(-80.07+0.60717\left(-500+T\right)\right)+\left(1/10\right)c^{2}\left(80.49-0.604377\left(-500+T\right)\right)}$$

Note that, in Equation (45), we have taken as correction factors

$h_{1}=h_{2}=h_{3}=h_{4}=1/10$, and $h_{5}=h_{6}=1/1000$, $h_{7}=h_{8}=1/100$, while, in Equation (46), we have taken $h_{1}=h_{2}=h_{3}=h_{4}=h_{5}=h_{6}=h_{7}=h_{8}=1/10$.

**Figure 1:**
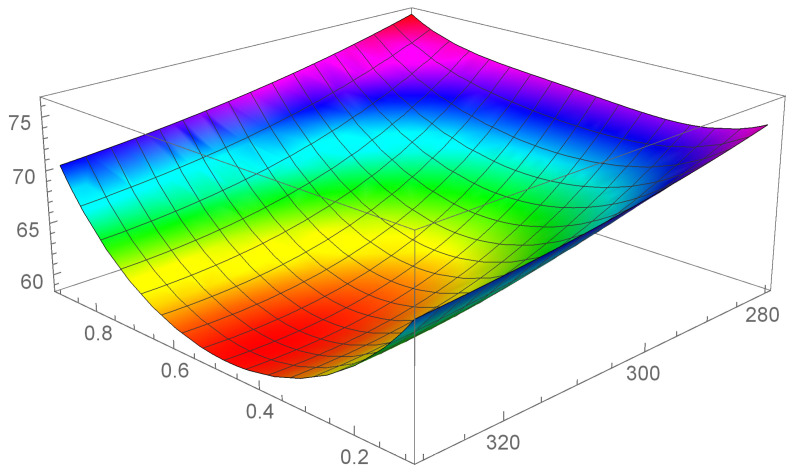
Heat conductivity as function of *c* and T, in the interval [T=280K–T=330K].

**Figure 2:**
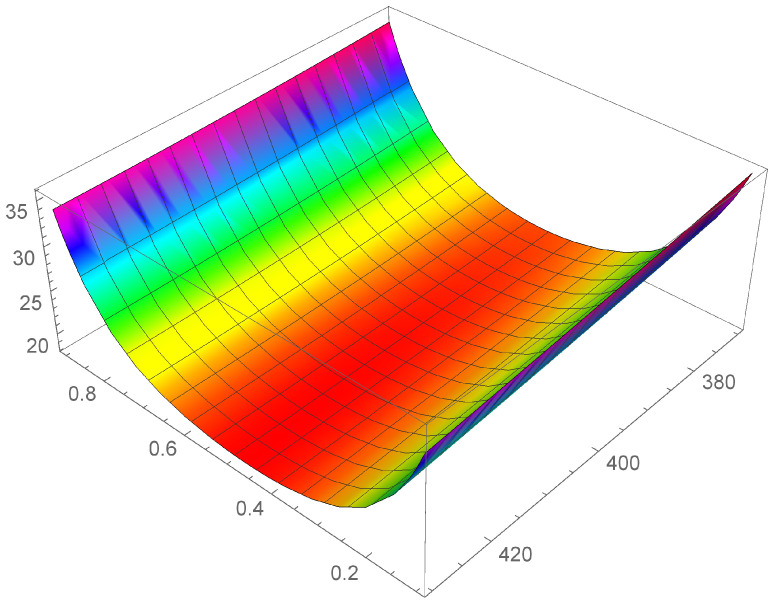
Heat conductivity as function of *c* and T, in the interval [T=380K–T=430K].

**Figure 3:**
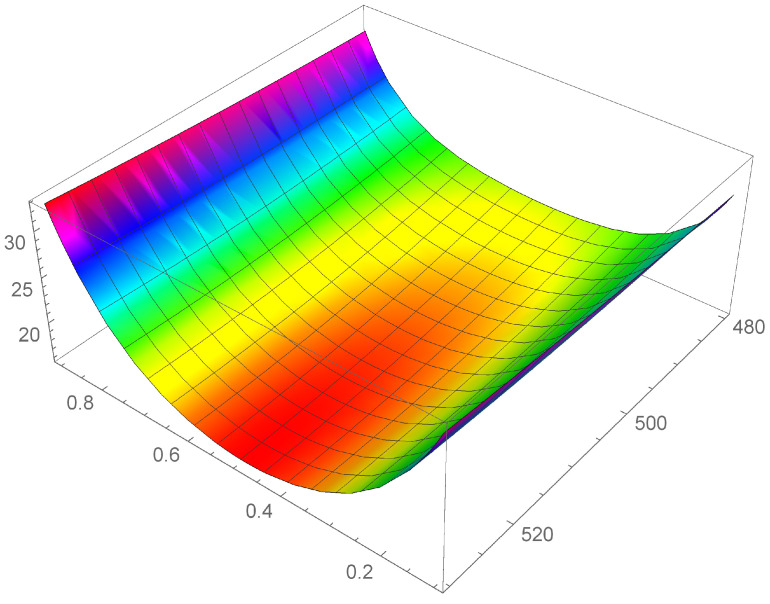
Heat conductivity as function of *c* and T, in the interval [T=480K–T=530K].

In Figure 1, Figure 2 and Figure 3 it is shown the plot of the function λ(c,T) in the intervals

[T=280K–T=330K], [T=380K–T=430K], and [T=480K–T=530K], respectively.

## 3. Results

For the rigid conductor considered here (L=3mm), in Ref. [18] we have calculated the minima of the rate on energy dissipated at a fixed operational temperature, by regarding such a rate as a function of the composition *c* and of *x* (see Equation (12)). In the present paper, instead, we carry on our analysis by supposing that *x* is fixed, while the rate of energy dissipated depends on composition *c* and temperature T, through the heat conductivity λ(c,T). To calculate the couples $\left(c_{opt},\;T_{opt}\right)$ which minimize E(c,T) around each temperature, we first observe that Equation (12) can now be rewritten as

(47) $$E\left(c,T\right)=k_{1}+k_{2}\;\lambda \left(c,T\right)$$

where

(48) $$k_{1}\equiv \frac{i^{2}}{\sigma_{e}}+i\left[\epsilon \;-\left(\Pi -\bar{E}\;\;\bar{l}\right)/T_{h}\right]\frac{T_{h}-T_{c}}{L}$$

and

(49) $$k_{2}\equiv \left[\left(1-b\right)/T_{h}\right]{\left(\frac{T_{h}-T_{c}}{L}\right)}^{2}$$

are constant. Then, the points of minimum of E(c,T) coincide with those of λ(c,T).

Before calculating these minima some preliminary considerations are in order.

First of all, we observe that by Figure 1, Figure 2 and Figure 3 it is evident that the heat conductivity, as function of composition, is more steep in the intervals 0≤c≤0.1 and 0.9≤c≤1. In fact, since the phonon scattering increases when a small amount of impurities of (Si in almost pure Ge, or Ge in almost pure Si), a subsequent reduction in thermal conductivity takes over for values of *c* close to the boundaries of the interval [0,1]. If, instead, the quantities of Ge and Si are almost comparable, no further appreciable reduction in λ takes over, so that λ is almost constant in the interval 0.1≤c≤0.9. Thus, we expect that $c_{opt}$ lies in this interval. Moreover, since we used a first-order series expansion of the material functions M(T), N(T), P(T), and Q(T) around one of the reference temperatures T=300K, T=400K, and T=500K, we look for $T_{opt}$ in the neighborhood of such temperatures. The considerations above are confirmed by the results in Table 4, wherein the values of $c_{opt}$, $T_{opt}$, and $\lambda_{opt}$ are reported for the three temperatures.

In order to give a first approximated estimation of the energy dissipated along a thermoelectric process, in Table 5 we show the calculated values of $E_{opt}$, corresponding to the three reference temperatures, for the system considered in [12]. To obtain such values, we supposed $T_{c}=T_{opt}$, and $T_{h}=T_{opt}+0.1K$, where $T_{opt}$ is the optimal temperature shown in Table 4, corresponding to the reference temperatures T=300K, T=400K, and T=500K, respectively. Such a small difference of temperature could appear unusual, but one must take into account that at very small scale even small difference of temperature produce very high gradients. Thus, our choice allows to obtain realistic values of $E_{opt}$. For the sake of calculation, we have approximated the optimal values of temperature by omitting the decimal digits. For ϵ and $\sigma_{e}$, we used the experimental data in Ref. [12]. As we observed above, in the genuinely non-linear regime the SKR is no longer true, in general [31]. Thus, in our computation, we used three different values of Π close but not equal to $\epsilon T_{h}$, namely $\Pi \left(325K\right)=2.8\times 10^{-2}$ V, $\Pi \left(425K\right)=4\times 10^{-2}$ V, and $\Pi \left(527K\right)=6\times 10^{-2}$ V. For $\bar{E}$ we have chosen the value of 1 V/m, and for $\bar{l}$ the value of 30nm. Such a value derives by the fact that in thermomass theory the value |l| is ever smaller of the mean free path of phonons in the system considered [25,26], and that such a value has been estimated to be around 40nm for Si–Ge alloys at room temperature [32]. For the material parameter b<1 entering the effective thermal conductivity, we have used the empirical value $1-b=10^{-2}$.

By the results above, we infer a substantial coincidence of the values of $c_{opt}$ in the neighborhood of the different temperatures. Furthermore, the difference $T_{opt}-T$ is almost the same for the three reference temperatures. We note that by the plots of λ emerges that it is maximum for pure crystals, i.e., in the presence of an ordered crystal lattice that is capable to reduce the phonon scattering. In the two dilute zones c∈[0,0.1] and c∈[0.9,1], which correspond to doped Ge and doped Si, respectively, λ starts to decrease (in [0.9,1]λ decreases going from Si to Ge), because the doping increases the phonon scattering. For c≃0.5, the ordered structure is lost, and the phonon scattering is maximum, due to the disordered spatial disposition of the molecules of the alloy. Thus, it is plausible to suppose that the higher the alloy disorder, the lower the thermal conductivity, and this explains why the minima of λ are all very close to c=0.5.

We note a discrepancy of the value of $\lambda_{opt}$ around T=300K with respect to the values around T=400K and T=500K. This is due to the fact that, as it can be seen by Table 1, the values of $\lambda_{\mathrm{Si}}$ and $\lambda_{\mathrm{Ge}}$ at T=300K are higher with respect to the values at T=400K and T=500K. This influences the corresponding values of the functions φ and γ and, as a consequence, the values of λ, as it is evident in Figure 1, Figure 2 and Figure 3. Finally, this difference in the values of λ, produces also a difference in the values of the rate of energy dissipated, as shown in Table 5.

**Figure 4:**
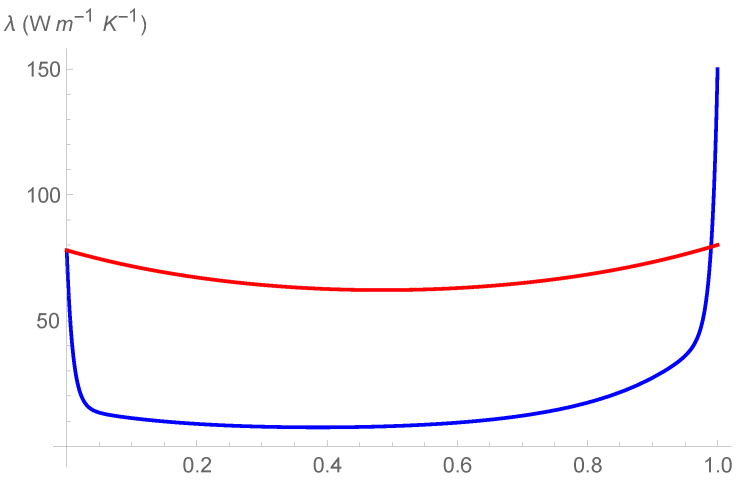
Comparison of different profiles of thermal conductivity at T=300K, as obtained in Ref. [18], (blue curve), and as obtained in the present investigation, (red curve).

**Figure 5:**
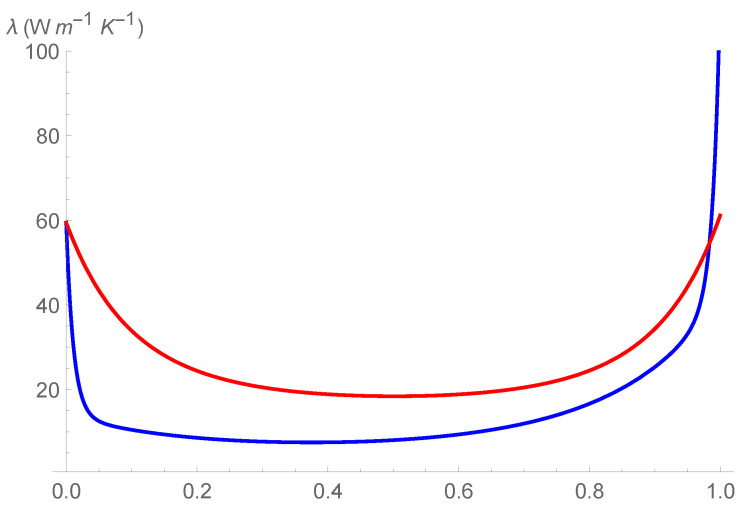
Comparison of different profiles of thermal conductivity at T=400K, as obtained in Ref. [18], (blue curve), and as obtained in the present investigation, (red curve).

**Figure 6:**
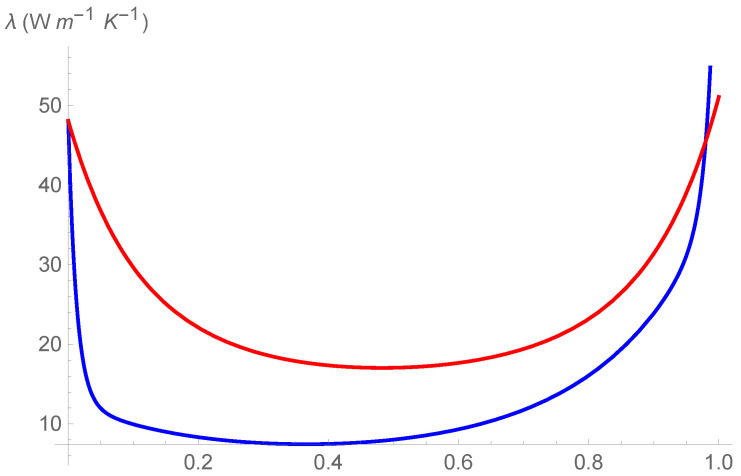
Comparison of different profiles of thermal conductivity at T=500K, as obtained in Ref. [18], (blue curve), and as obtained in the present investigation, (red curve).

For the sake of comparison, in Figure 4, Figure 5 and Figure 6, we show the curves λ=λ(c), as obtained in Ref. [18], (blue curve) and λ=λ(c,300K), as obtained in the present investigation (red curve). Doing that, one should take into account that the red curves do not follow by a fit of λ, but they follow by a first-order approximation of λ as function of T. Perhaps, such an approximation can be improved and the differences between the two curves can be reduced. We aim at doing that in future investigations.

We can observe the two following marked differences:

- The values of λ=λ(c,300K) are higher of the values of λ=λ(c), even in the dilute intervals 0≤c≤0.1 and 0.9≤c≤1;
- In two narrow intervals close to c=0 and c=1, the curve λ=λ(c,300K) is less steep of the curve λ=λ(c).

Thus, in the composition and temperature dependent representation of λ, the effect of phonon scattering in the decrement of λ is still present but is less dramatic, even for doped Si and doped Ge. We will test further such a result in next investigations.

The analysis developed in the present research yields new information on how manufacturing homogeneous thermoelectric generators made by Silicon-Germanium alloys, by determining the composition and the operational temperature which optimize their efficiency. In fact, we may conclude that:

- Around T=300K, the optimal thermoelectric energy conversion is achieved at T=325.268K in a homogeneous silicon–germanium wire ${\mathrm{Si}}_{c}{\mathrm{Ge}}_{1-c}$, with c=0.492488;
- Around T=400K, the optimal thermoelectric energy conversion is achieved at T=425.473K in a homogeneous silicon–germanium wire ${\mathrm{Si}}_{c}{\mathrm{Ge}}_{1-c}$, with c=0.479984;
- Around T=500K, the optimal thermoelectric energy conversion is achieved at T=527.669K in a homogeneous silicon–germanium wire ${\mathrm{Si}}_{c}{\mathrm{Ge}}_{1-c}$, with c=0.49022.

Our next investigation will be devoted to the following tasks:

- To improve the approximation developed here, perhaps using a second-order series expansion;
- To look for experimental data on the dependency of Seebeck coefficient and thermal conductivity on the composition, in order to obtain a fit also for those quantities;
- To look for more data concerning the dependency of the material functions considered here on temperature, and try to get a fit as well.

In further researches, we also aim at investigating the same type of problem considered here, if the constitutive equation for the heat flux changes in

(50) $$q=-\lambda \nabla T+\mu q\cdot \nabla q+\ell^{2}\left(\Delta q+\nabla \nabla \cdot q\right)+\Pi i$$

where μ is a material function and *ℓ* is the mean free path of the phonons, which in solid crystals are responsible of the heat transport. The previous constitutive relation follows by a non-linear generalization of the celebrated Guyer–Krumhansl equation [33], which, from the macroscopic point of view, can be obtained within the framework of Extended Irreversible Thermodynamics [7,34,35]. In addition to non-linear effects, represented by the non-linear dependency of λ on composition and temperature and by the term μq·∇q, Equation (50) takes also into account non-local interactions of phonons through the additional term $\ell^{2}\left(\Delta q+2\nabla \nabla \cdot q\right)$. In general, such a term is negligible for bulk systems, as that analyzed in the present paper, but becomes important in nanosystems, where the characteristic length of the system is comparable to the mean free path of the heat carriers [7,27,28]. Thus, at nanometric scale, it would be interesting to analyze the problem considered here by using Equation (50) instead of Equation (8), in order to investigate the influence of non-local effects on the efficiency of thermoelectric energy conversion.

## Figures and Tables

**Table 1:** Results for $\left(\lambda_{opt},\;c_{opt}\right)$ in Ref. [18], for L=3mm.

Temperature (K)	$c_{opt}$	$\lambda_{opt}$ (W m${}^{-1}$ K${}^{-1}$)
T=300	0.385989	7.51235
T=400	0.375079	7.48291
T=500	0.36537	7.42273

**Table 2:** Thermal conductivity of pure Si and pure Ge, for a wire of length L=3mm (Ref. [18]).

Temperature (K)	$\lambda_{\mathrm{Si}}$ (W m${}^{-1}$ K${}^{-1}$)	$\lambda_{\mathrm{Ge}}$ (W m${}^{-1}$ K${}^{-1}$)
T=300	149.95	77.95
T=400	113.54	59.42
T=500	92.01	48.08

**Table 3:** The material parameters *M*, *N*, *P*, and *Q* in Equation (2) for a ${\mathrm{Si}}_{c}{\mathrm{Ge}}_{1-c}$ wire of length L=3mm (see Ref. [18]).

Temperature (K)	*M*	*N*	*P*	*Q*
T=300	4.8706	−3.76	109.452	−108.953
T=400	91.804	−91.351	4.416	−3.3127
T=500	80.4998	−80.0781	4.0667	−2.9717

**Table 4:** Values of $c_{opt}$, $T_{opt}$, and $\lambda_{opt}$ for L=3mm.

Temperature (K)	$c_{opt}$	$T_{opt}$ (K)	$\lambda_{opt}$ (W m${}^{-1}$ K${}^{-1}$)
T=300	0.492488	325.268	58.7111
T=400	0.479984	425.473	18.2765
T=500	0.49022	527.669	15.8442

**Table 5:** Values of $E_{opt}$ calculated by using Equation (12) and the experimental data for the system considered in Ref. [12].

Temperature (K)	$E_{opt}$ (W m${}^{-3}$)
T=300	2.84474
T=400	1.20818
T=500	1.02397

## Data Availability

Not applicable.

## References

[1] Carlomagno I., Cimmelli V.A., Jou D. (2016). Computational analysis of heat rectification in composition-graded systems: From macro-to-nanoscale. Physica B.

[2] Kuznetsov V.L., Rowe D.M. (2005). Functionally Graded Materials for Termoelectric Applications.

[3] Nolas G.S., Sharp J., Goldsmid H.J. (2001). Thermoelectrics: Basic Principles and New Materials Developments.

[4] Lebon G., Jou D., Casas-Vázquez J. (2008). Understanding Nonequilibrium Thermodynamics.

[5] Feldhoff A. (2020). Power Conversion and Its Efficiency in Thermoelectric Materials. Entropy.

[6] Feldhoff A. (2022). On the Thermal Capacity of Solids. Entropy.

[7] Sellitto A., Cimmelli V.A., Jou D. (2016). Mesoscopic Theories of Heat Transport in Nanosystems.

[8] Jou D., Casas-Vázquez J., Lebon G. (2010). Extended Irreversible Thermodynamics, 4th revised ed..

[9] Li D., Wu Y., Kim P., Shi L., Yang P., Majumdar A. (2003). Thermal conductivity of individual silicon nanowires. Appl. Phys. Lett..

[10] Joshi G., Lee H., Wang Y.L., Zhu G.X., Wang D., Gould R.W., Cuff D.C., Tang M.Y., Dresselhaus M.S., Chen G. (2008). Enhanced Thermoelectric Figure-of-Merit in Nanostructured p-type Silicon Germanium Bulk Alloys. Nano Lett..

[11] Raphael A., Singh A.K., Vivekanandhan P., Kumaran S. (2021). Thermoelectric performance of nanostructured PbSnTeSe high entropy thermoelectric alloy synthesized via spark plasma sintering. Physica B.

[12] Wongprakarna S., Pinitsoontornb S., Tanusilpd S., Kurosaki K. (2018). Enhancing thermoelectric properties of p-type SiGe alloy through optimization of carrier concentration and processing parameters. Mater. Sci. Semicond. Process..

[13] Cimmelli V.A., Rogolino P., Sellitto A. (2016). A nonlinear model of thermoelectricity with two temperatures: Application to quasicrystalline nanowires. J. Math. Phys..

[14] Rogolino P., Sellitto A., Cimmelli V.A. (2017). Minimal Entropy Production and Efficiency of Energy Conversion in Nonlinear Thermoelectric Systems with Two Temperatures. J. Non-Equilib. Thermodyn..

[15] Rogolino P., Cimmelli V.A. (2018). Thermoelectric efficiency of graded Si*_c_* Ge_1−_*_c_* alloys. J. Appl. Phys..

[16] Rogolino P., Cimmelli V.A. (2020). Fitting thermal conductivity and optimizing thermoelectric efficiency of functionally graded Si*_c_* Ge_1−_*_c_* nanowires. Math. Comput. Simul..

[17] Rogolino P., Cimmelli V.A. (2020). Thermal conductivity and enhanced thermoelectric efficiency of composition graded Si*_c_* Ge_1−_*_c_* alloys. Z. Angew. Math. Phys..

[18] Rogolino P., Cimmelli V.A. (2020). Thermoelectric efficiency of Silicon-Germanium alloys in Finite Time Thermodynamics. Entropy.

[19] Cimmelli V.A., Rogolino P. (2022). New and Recent Results for Thermoelectric Energy Conversion in Graded Alloys at Nanoscale. Nanomaterials.

[20] Glassbrenner C., Slack G. (1964). Thermal conductivity of silicon and germanium from 3° K to the melting point. Phys. Rev..

[21] Steele M., Rosi F. (1958). Thermal conductivity and thermoelectric power of germanium-silicon alloys. J. Appl. Phys..

[22] Abeles B., Beers D., Cody G., Dismukes J. (1962). Thermal conductivity of Ge-Si alloys at high temperatures. Phys. Rev..

[23] Caim J.W., Bell E. (2014). Mathematics of Fitting Sicientific Data.

[24] Walstrom P.L. (1988). Spatial dependence of thermoelectric voltages and reversible heats. Am. J. Phys..

[25] Sellitto A., Cimmelli V.A. (2012). A continuum approach to thermomass theory. ASME J. Heat Transf..

[26] Sellitto A., Cimmelli V.A. (2014). Flux Limiters in Radial Heat Transport in Silicon Nanolayers. J. Heat Transf..

[27] Lebon G. (2014). Heat conduction at micro and nanoscales: A review through the prism of Extended Irreversible Thermodynamics. J. Non-Equilib. Thermodyn..

[28] Jou D., Cimmelli V.A. (2016). Constitutive equations for heat conduction in nanosystems and nonequilibrium processes: An overview. Comm. Appl. Ind. Math..

[29] Dong Y., Cao B.-Y., Guo Z.-Y. (2011). Generalized heat conduction laws based on thermomass theory and phonon hydrodynamics. J. Appl. Phys..

[30] Wang M., Yang N., Guo Z.-Y. (2011). Non-Fourier heat conductions in nanomaterials. J. Appl. Phys..

[31] Rogolino P., Sellitto A., Cimmelli V.A. (2015). Influence of nonlinear effects on the efficiency of a thermoelectric generator. Z. Angew. Math. Phys..

[32] Carlomagno I., Cimmelli V.A., Jou D. (2020). Tunable heat rectification by applied mechanical stress. Phys. Lett. A.

[33] Guyer R.A., Krumhansl J.A. (1966). Solution of the linearized phonon Boltzmann equation. Phys. Rev..

[34] Cimmelli V.A. (2009). Different thermodynamic theories and different heat conduction laws. J. Non-Equilib. Thermodyn..

[35] Cimmelli V.A., Sellitto A., Jou D. (2010). Nonequilibrium temperatures, heat waves, and nonlinear heat transport equations. Phys. Rev. B.

